# A Reinforcement Learning-Based Framework for Tariff-Aware Load Shifting in Energy-Intensive Manufacturing

**DOI:** 10.3390/s26061858

**Published:** 2026-03-15

**Authors:** Jersson X. Leon-Medina, Mario Eduardo González Niño, Claudia Patricia Siachoque Celys, Bernardo Umbarila Suarez, Francesc Pozo

**Affiliations:** 1 Grupo de Investigación en Biochar, Sueloy Cambio Climático (Pyrosfera), Suministros Mineros e Industriales de Colombia LTDA-Sumininco LTDA, Km1 vía Nobsa-Duitama Vereda Guaquida, Nobsa 152280, Colombia; cpsiachoque@gmail.com (C.P.S.C.); bernardoumbarila@gmail.com (B.U.S.); 2 Escuela de Ingeniería Electromecánica, Facultad Seccional Duitama, Universidad Pedagógica y Tecnológica de Colombia, Carrera 18 con Calle 22, Duitama 150461, Colombia; marioeduardo.gonzalez@uptc.edu.co; 3 Control, Data, and Artificial Intelligence (CoDAlab), Department of Mathematics, Escola d’Enginyeria de Barcelona Est (EEBE), Campus Diagonal-Besòs (CDB), Universitat Politècnica de Catalunya (UPC), Eduard Maristany 16, 08019 Barcelona, Spain; 4 Institute of Mathematics (IMTech), Universitat Politècnica de Catalunya (UPC), Pau Gargallo 14, 08028 Barcelona, Spain

**Keywords:** reinforcement learning, Proximal Policy Optimization, energy management systems, smart scheduling, time-of-use tariffs, industrial IoT, energy efficiency, limeindustry

## Abstract

Optimizing energy-intensive manufacturing under time-varying electricity tariffs requires scheduling strategies that reduce cost without compromising operational feasibility. This study is grounded in readily available industrial sensing: we exclusively use time-series measurements of aggregated active power and energy at the main distribution board of a quicklime production plant. We propose a tariff-aware load-shifting framework in which a Proximal Policy Optimization (PPO) reinforcement learning agent is trained in a custom Gymnasium environment to apply discrete consumption scaling actions constrained to 80–125% of a baseline profile during the operating shift (08:00–16:00), explicitly accounting for demand-charge exposure in the TOU peak window (13:00–15:00). The reward design combines instantaneous electricity cost with cumulative energy-tracking penalties and terms associated with operational constraints. Multi-day validation over N=30 working days shows consistent economic benefits, with a median total cost reduction on the order of 10% (narrow IQR) driven by reduced peak-window energy and demand peaks. However, the script-based binary compliance indicators (viol_energy, viol_prod_min) reveal deviations from the energy-balance criterion and occasional minimum-production shortfalls under the tolerances used, highlighting the cost–production trade-off and the need for stricter constraint handling for industrial deployment. In addition, we benchmark against dynamic programming (DP), an alternative RL policy (DQN), and a greedy heuristic (GREEDY), comparing cost; operational performance; and, when applicable, computational efficiency, which positions PPO as a competitive alternative among the considered methods. Overall, this work demonstrates how learning-based decision making can be coupled with real-world industrial sensing infrastructures, providing a data-driven tariff-aware scheduling layer for industrial energy management under practical constraints.

## 1. Introduction

The increasing hourly variability of electricity prices, driven by dynamic tariff schemes and growing energy demand, has motivated the development of tariff-aware scheduling strategies. These strategies aim to minimize operational costs without compromising the continuous operation of industrial processes [1,2]. In energy-intensive sectors, such as lime production, avoiding the operation of high-power equipment during peak tariff windows is essential, as electricity costs directly impact overall operational margins [1]. This challenge is further amplified by the ongoing evolution of power systems towards more decentralized, digitalized, and renewable-rich architectures, where operational uncertainty and real-time decision-making requirements increase substantially [3,4,5]. As a result, scheduling in modern electricity markets becomes not only a cost minimization problem, but also a robustness problem under dynamic, partially predictable conditions [6,7].

While demand forecasting techniques allow for the construction of expected consumption profiles to plan operations, converting these profiles into sequential decision-making actions poses significant challenges. This is particularly true under strict technical constraints and multi-objective scenarios [8,9]. In practice, industrial scheduling must often account for tariff structures that go beyond classical time-of-use (TOU), including real-time pricing, critical peak pricing, threshold-based penalties, and demand charges, which complicates the design of policies that remain cost-effective and operationally feasible [6]. In this context, reinforcement learning (RL) has emerged as a powerful tool for solving sequential optimization problems without requiring an explicit mathematical model of the environment. Its ability to learn policies that balance multiple objectives under uncertainty makes it ideal for industrial applications [10,11]. Moreover, deep reinforcement learning (DRL) has been increasingly positioned as a data-driven alternative when classical optimization and control approaches become brittle under non-stationary conditions, uncertainty, and high-dimensional decision spaces [3,4].

Recent studies have demonstrated the viability of RL for energy scheduling in systems subject to time-of-use (ToU) tariffs. In these settings, decisions must optimize energy consumption by considering hourly price fluctuations, operational limits, and production targets [12]. Such approaches have been successfully applied in diverse contexts, including biogas plants [9], electric vehicle charging stations [11,13], and building demand response programs where price signals are used to shape consumption behavior [14]. Importantly, this growing body of work highlights that price-driven scheduling problems are often shaped by three practical features: (i) uncertain and time-varying market signals, (ii) heterogeneous and constrained loads, and (iii) the need for fast decision making compatible with real operational timelines [6,7,13]. Furthermore, the integration of RL allows for the incorporation of autonomous control features that align with Industry 4.0 trends, where algorithms can manage operational constraints, customized penalties, and energy efficiency criteria simultaneously [15,16]. This direction is consistent with broader DRL applications across modern energy-internet frameworks, spanning demand response, market participation, resilience, and the coordination of distributed resources [3,5].

At the industrial level, the decarbonization of energy-intensive processes increasingly demands strategies that exploit operational flexibility and participate in demand response (DR) without compromising production continuity [17,18]. However, industrial environments impose strong constraints (process coupling, discrete/continuous operational modes, and production targets) that limit flexibility and make purely rule-based or rigid optimization approaches difficult to sustain under changing tariffs [6,17]. This motivates the adoption of learning-based scheduling policies that can adapt online to uncertainty while respecting process requirements, and also opens pathways to more structured extensions (e.g., decentralized or multi-agent coordination mechanisms) when multiple assets must cooperate [17,19]. At the same time, prior studies in other energy domains underline that DRL can handle complex couplings and uncertainties (e.g., multi-energy electricity–heat–hydrogen systems or geographically dependent hydrogen infrastructure planning), reinforcing the relevance of DRL as a general decision-making paradigm under uncertainty [20,21].

Despite these advantages, practical deployment of DRL faces well-known barriers, including reward design, explicit constraint handling, data quality and availability, and the gap between simulation performance and real-world operation [3,6]. Additionally, “unsafe” exploration can incur high costs during early learning stages, which can undermine economic feasibility [14]. Risk-aware strategies—such as combining model-free learning with planning environments and guardrails to limit costly actions—have been proposed as a way to mitigate initial learning costs, suggesting an important direction for operationally safe scheduling in real applications [14]. These insights provide useful motivation for industrial tariff-aware scheduling, where early-stage exploration can be expensive and constraints are strict.

This article proposes a tariff-aware energy scheduling framework based on deep reinforcement learning (DRL) applied to the aggregated consumption of an industrial lime plant. Using a Proximal Policy Optimization (PPO) agent, the system determines the optimal hourly consumption fraction within a work shift. The objective is to shift energy-intensive loads away from peak hours without compromising the total daily energy required for production. This approach contributes to the digital transformation of traditional industries by providing a scalable, data-driven methodology for cost-effective and sustainable operation. The use of DRL methods such as PPO is further motivated by their suitability for complex tariff environments and sequential decision making, which have been highlighted in recent DRL-based energy management studies under dynamic pricing and operational constraints [6,7]. Moreover, insights from demand response applications suggest that incorporating behavioral uncertainty, heterogeneity, and risk-awareness can be key for robust, deployable decision policies [14,19,22].

The remainder of this paper is organized as follows: Section 2 shows TOU-aware scheduling related work. Section 3.1 describes the industrial context and the dataset used in this study, together with the construction of the baseline load profile. Section 3.3 presents the mathematical formulation of the tariff-aware scheduling problem and details the reinforcement learning environment, including the state and action spaces, reward structure, and training configuration. Section 4 reports and analyzes the experimental results obtained with the proposed approach. Finally, Section 5 concludes the paper by summarizing the main findings and outlining directions for future research.

## 2. Related Work

Time-of-use (TOU) tariffs have motivated a growing body of research on reinforcement learning (RL) for tariff-aware energy scheduling, where the goal is to learn sequential decision policies that translate time-varying price signals and operational states into feasible actions over a finite horizon. In this literature, deep reinforcement learning (DRL) has gained prominence in settings with large decision spaces and realistic operational constraints. Among actor–critic methods, Proximal Policy Optimization (PPO) is frequently adopted for tariff-aware control due to its training stability and robustness in practical implementations, and it has been reported in applications including electric vehicle (EV) charging infrastructure, microgrids, and grid-interactive systems [23,24]. Comparative analyses also highlight PPO and neuroevolutionary RL approaches as competitive alternatives in multi-objective energy optimization problems [25].

Despite these advances, the dominant empirical evidence remains concentrated in a limited set of domains, particularly EV charging and building energy management. In EV charging, RL formulations commonly incorporate network constraints, voltage stability considerations, fairness objectives, and decentralized or multi-agent coordination, under the assumption of fine-grained device-level monitoring and controllability [23,26]. In buildings, DRL controllers are typically designed to shift flexible loads in response to TOU tariffs and system-level objectives, relying on relatively high operational flexibility and detailed instrumentation [27,28]. These assumptions do not necessarily hold in industrial manufacturing.

Industrial environments introduce structural differences that reshape the scheduling problem. First, sensing infrastructures often provide only aggregated measurements at the main distribution board (point of common coupling), restricting control actions to plant-level demand rather than device-level actuation. Second, production processes are tightly coupled and flexibility is often limited, so TOU-aware scheduling must explicitly account for production feasibility and operational continuity. Under these conditions, a recurring gap in RL-based tariff-aware scheduling is the limited emphasis on enforcing daily energy equivalence and production targets through mechanisms such as cumulative tracking terms and terminal constraints, which are critical when deferring consumption can lead to infeasible end-of-shift recovery. Moreover, several existing frameworks tightly couple forecasting and control, making it difficult to isolate the economic effect of scheduling decisions from prediction accuracy.

In this context, the present study positions itself in TOU-aware scheduling of an aggregated industrial load representative of a quicklime production plant, with an explicit focus on main-board sensing assumptions and daily energy and production feasibility. The contribution is framed around: (i) a finite-horizon formulation compatible with aggregated measurements, where actions operate as discrete scaling of a baseline profile; (ii) an explicit energy–production link to represent operational constraints beyond cost; (iii) an incentive structure including cumulative tracking and terminal penalties to promote daily energy equivalence and preservation of production targets; (iv) an evaluation scheme based on comparison against a baseline using indicators aligned with industrial operation (total cost, peak exposure, and production-normalized metrics), including multi-day validation to assess consistency; and (v) the use of real industrial hourly data, enabling discussion under realistic instrumentation limitations and suggesting natural extensions toward stronger constraint guarantees, multi-day horizons, and explicit treatment of uncertainty in tariffs and baseline profiles.

## 3. Materials and Methods

### 3.1. Dataset and Industrial Context

The experimental evaluation uses a real-world dataset obtained from electrical consumption measurements collected at an industrial quicklime production plant located in Colombia. The dataset reflects actual operational conditions of the facility and captures the intrinsic variability associated with industrial production processes, including load fluctuations, fixed operating schedules, and exposure to time-of-use electricity tariffs. The dataset was obtained from the facility’s existing energy monitoring infrastructure at the main distribution board (PCC). The study used historical hourly measurements retrieved from the deployed smart meter and communication modem; no additional sub-metering or equipment-level sensors were installed for this work.

The experimental dataset used in this study was obtained from a real industrial energy monitoring system installed at the main electrical distribution board of the quicklime production facility [29]. Active power consumption was measured using an ACTARIS/ITRON AC6000 (Itron, Inc., Liberty Lake, WA, USA) smart energy meter, accuracy class 0.5S, rated at 5(10) A and 57–240/415 V (±20%), operating in bidirectional mode and supporting both two- and three-element self-powered configurations. The meter provides dual load-curve recording capabilities with internal memory channels and communicates through RS-232 and IEC 61107 optical interfaces. Remote data acquisition was enabled via a ROBUSTEL M1201 (Robustel Technologies Co., Ltd., Guangzhou, China) industrial 4G communication modem, supporting secure encrypted data transmission over serial RS-232/RS-485 interfaces. The communication unit was deployed with an external antenna and dedicated power supply, ensuring stable and continuous operation under industrial conditions.

Electrical consumption data were recorded at an hourly resolution and correspond to the standard daytime production shift of the plant, which operates from 08:00 to 16:00. Outside this operating window, including nighttime hours and weekends, the recorded electrical demand is zero, as no production activity takes place.

The primary variable considered in this study is the aggregated electrical power consumptionmeasured at the main electrical distribution board of the plant. As a result, the proposed scheduling strategy operates at the plant level rather than at the level of individual motors or production units. This choice reflects the practical limitations of industrial sensing infrastructures, where smart energy meters or power analyzers typically provide aggregated measurements at the point of common coupling, while detailed equipment-level monitoring may be unavailable or economically impractical.

**Figure 1:**
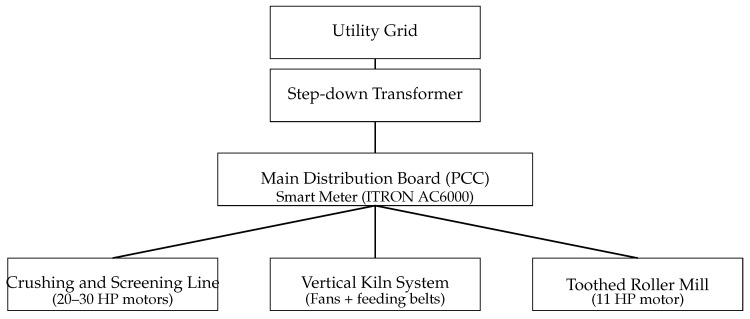
Simplified pseudo single-line electrical diagram of the SUMININCO quicklime production plant. Electrical measurements used by the reinforcement learning controller are acquired at the main distribution board (PCC), where aggregated plant demand is recorded.

To provide additional technical context regarding the studied industrial system, Table 1 summarizes the main electrically driven equipment involved in the quicklime production process at the SUMININCO facility, including installed motor power ratings, load types, and their functional roles within the process line. In addition, Figure 1 presents a simplified pseudo single-line electrical diagram of the plant, highlighting the main distribution board (PCC) where aggregated electrical measurements are acquired.

Although the reinforcement learning controller operates exclusively on aggregated electrical measurements at the PCC, the equipment listed in Table 1 represents the dominant AC motor loads that define the plant-level demand profile observed by the smart meter.

The main electrically driven equipment listed in Table 1 represents an installed motor capacity of approximately 102 HP (≈76 kW), dominated by continuous AC motor loads associated with material handling, crushing, and screening operations. These loads define the primary electrical demand profile measured at the main distribution board.

From a sensing perspective, the proposed methodology assumes a non-intrusive monitoring architecture based on plant-level electrical sensors commonly deployed in industrial energy management systems. The state representation used by the reinforcement learning agent is constructed from normalized features directly derived from these sensor measurements, including instantaneous power demand, cumulative energy consumption, and their temporal evolution over the operating shift. This design ensures that the learning agent operates exclusively on physically measured sensor data, avoiding reliance on simulated signals, virtual meters, or equipment-specific models, thereby enhancing practical deployability in real industrial environments.

From a control and deployment perspective, the aggregated-load formulation offers two main advantages. First, it enables the proposed reinforcement learning-based scheduling framework to be integrated directly with existing industrial energy monitoring systems, without requiring additional sensors or intrusive modifications to the production process. Second, it allows the scheduling policy to implicitly capture the collective behavior of multiple alternating current (AC) motors that dominate the plant’s electrical demand, while maintaining a tractable and scalable decision-making problem.

The baseline load profile used in the experiments represents the expected hourly consumption under nominal operating conditions and is constructed directly from historical measurements. This baseline reflects typical intra-shift consumption patterns observed during normal production and serves as the reference trajectory for enforcing daily energy conservation and production equivalence. The focus of this work is therefore not on improving load forecasting accuracy, but on exploiting operational flexibility in real measured demand profiles to achieve optimal temporal reallocation of energy consumption under time-varying electricity tariffs and industrial constraints.

### 3.2. Baseline Load Profile Construction

The reinforcement learning agent operates on an expected daily load profile, hereafter referred to as the baseline. This baseline represents the anticipated electrical demand of the plant over the working shift (08:00–16:00) under nominal operating conditions and serves as the reference trajectory for tariff-aware scheduling.

The baseline profile is constructed from aggregated historical consumption patterns of the plant and subsequently smoothed to capture the typical intra-shift variability of alternating current (AC) motors, which constitute the dominant electrical loads in the process. As such, the baseline reflects realistic production-driven demand dynamics while filtering high-frequency fluctuations that are not relevant at the hourly scheduling resolution considered in this study.

In the experimental setup reported in this work, the baseline profile constitutes the sole exogenous input to the reinforcement learning environment. The scheduling agent neither performs online load forecasting nor updates the baseline during an episode. Instead, the baseline is interpreted as a fixed expected demand curve provided by an upstream planning or forecasting module, consistent with common industrial practice where production schedules are defined in advance of execution.

Consequently, classical forecasting performance metrics such as the mean absolute error (MAE) or root mean square error (RMSE) are not reported in this study. The objective of the proposed approach is not to improve demand prediction accuracy but to optimally reallocate an expected load profile in time in response to time-of-use electricity tariffs and operational constraints. The performance evaluation therefore focuses on cost reduction, peak load mitigation, and production preservation resulting from the scheduling policy.

It is important to note that the proposed framework is modular by design. The baseline profile can be readily replaced by the output of any forecasting model—including statistical, machine learning, or deep learning approaches—without modifying the reinforcement learning formulation. This decoupling between prediction and decision making enables flexible integration of the proposed scheduler into existing energy management systems and facilitates future extensions incorporating forecast uncertainty or adaptive baseline updates.

### 3.3. Mathematical Formulation of the Tariff-Aware Scheduling Problem

This section presents the formal mathematical formulation of the proposed tariff-aware scheduling problem with explicit production modeling, suitable for reinforcement learning-based control of energy-intensive industrial processes.

#### 3.3.1. Time Indexing and Baseline Data

We consider a single working day discretized into H=9 hourly time steps, corresponding to the operating window from 08:00 to 16:00. Each time step is indexed by t∈{0,…,H−1}.

The baseline electrical power consumption profile, measured at the main distribution board, is denoted by

(1) $${\left\{b_{t}\right\}}_{t=0}^{H-1},\;b_{t}\geq 0\;\left(\mathrm{kW}\right),$$

which represents the expected power demand under nominal operation without tariff-aware control.

The time-dependent electricity price is defined as

(2) $${\left\{p_{t}\right\}}_{t=0}^{H-1},\;p_{t}\geq 0\;\left(\mathrm{currency}/\mathrm{kWh}\right).$$

Let $h_{t}=8+t$ denote the clock hour associated with time step *t*, with t∈{0,…,H−1}. The peak tariff window is defined as

(3) $$P=\{t|h_{t}\in \left\{13,14\right\}\},$$

corresponding to 13:00–15:00 at hourly resolution.

#### 3.3.2. Hourly Control Action

At each time step *t*, the reinforcement learning agent selects a discrete scaling factor

(4) $$a_{t}\in A=\left\{0.80,\;0.90,\;1.00,\;1.10,\;1.25\right\},$$

representing the fraction of the baseline load to be scheduled at that hour.

The resulting applied power is given by

(5) $$u_{t}=\mathrm{clip}\left(a_{t}b_{t},\;\underline{\lambda }b_{t},\;\bar{\lambda }b_{t}\right),$$

where $\underline{\lambda }=0.80$ and $\bar{\lambda }=1.25$ define the minimum and maximum admissible operational load, respectively. These bounds reflect industrial constraints related to minimum production continuity and allowable short-term overloading.

#### 3.3.3. Daily Energy Accounting

The total baseline daily energy requirement is

(6) $$E_{*}=\sum_{t=0}^{H-1}b_{t},$$

while the energy scheduled by the agent is

(7) $$E=\sum_{t=0}^{H-1}u_{t}.$$

The cumulative baseline energy up to time *t* is defined as

(8) $$B_{t}=\sum_{k=0}^{t}b_{k}.$$

#### 3.3.4. Explicit Production Modeling

To explicitly link electrical consumption with industrial output, we introduce a load-dependent production efficiency model.

The normalized load ratio is defined as

(9) $$\ell_{t}=\left\{\begin{array}{cc} \frac{u_{t}}{b_{t}}, & b_{t}>0,\\ 0, & b_{t}=0. \end{array}\right.$$

The efficiency as a function of load is modeled as

(10) $$\eta \left(\ell \right)=\eta_{\min}+\left(\eta_{\max}-\eta_{\min}\right)\ell^{\rho },$$

where $\eta_{\min}$ and $\eta_{\max}$ denote the minimum and maximum achievable efficiencies, and ρ controls the nonlinearity of the efficiency curve.

Hourly production is computed as

(11) $${\mathrm{prod}}_{t}=\alpha \;u_{t}\;\eta \left(\ell_{t}\right),$$

where α is a conversion factor relating electrical energy to material output (e.g., ton/kWh).

Total daily production is therefore

(12) $$\mathrm{PROD}=\sum_{t=0}^{H-1}{\mathrm{prod}}_{t}.$$

A minimum required production level is imposed as

(13) $${\mathrm{PROD}}_{\min}=\gamma \;{\mathrm{PROD}}_{*},$$

where γ∈(0,1] is a tolerance factor and

(14) $${\mathrm{PROD}}_{*}=\sum_{t=0}^{H-1}\alpha \;b_{t}\;\eta \left(1\right),$$

represents the reference production under baseline operation.

#### 3.3.5. Electricity Cost Modeling

The total energy cost over the day is computed as

(15) $$C_{\mathrm{ene}}=\sum_{t=0}^{H-1}u_{t}\;p_{t}.$$

In addition, a demand charge based on the maximum power during the peak tariff window is defined as

(16) $$\mathrm{peak}=\max_{t\in P}u_{t},$$

leading to a demand cost

(17) $$C_{\mathrm{dem}}=\kappa \cdot \mathrm{peak},$$

where κ is a tariff-dependent demand coefficient.

The total electricity-related cost is thus

(18) $$C_{\mathrm{tot}}=C_{\mathrm{ene}}+C_{\mathrm{dem}}.$$

#### 3.3.6. Energy Tracking Term

To prevent excessive deferral of energy consumption toward the end of the day, a normalized tracking error is defined as

(19) $${\mathrm{trk}}_{t}=\frac{\left(E_{t-1}+u_{t}\right)-B_{t}}{E_{*}},$$

where

(20) $$E_{t-1}=\sum_{k=0}^{t-1}u_{k}$$

is the cumulative scheduled energy prior to time *t*.

#### 3.3.7. Reward Function

The instantaneous reward at time *t* is defined as

(21) $$r_{t}=-u_{t}p_{t}-\beta_{\mathrm{trk}}\;{\left({\mathrm{trk}}_{t}\right)}^{2},$$

where

- $u_{t}$ denotes the scheduled electrical power at time step *t* (kW);
- $p_{t}$ is the normalized electricity price at time step *t* (cost units/kWh);
- ${\mathrm{trk}}_{t}$ represents the cumulative energy tracking error up to time *t*;
- $\beta_{\mathrm{trk}}$ is a weighting coefficient for energy tracking.

Terminal penalties applied at the end of the episode include

(22) $$\begin{array}{cc} R_{\mathrm{ener}} & =-\lambda_{\mathrm{ener}}{\left(\frac{E-E_{*}}{E_{*}}\right)}^{2}, \end{array}$$

(23) $$\begin{array}{cc} R_{\mathrm{prod}} & =-\lambda_{\mathrm{prod}}{\left(\max\;\left(0,\frac{{\mathrm{PROD}}_{\min}-\mathrm{PROD}}{{\mathrm{PROD}}_{\min}}\right)\right)}^{2}, \end{array}$$

(24) $$\begin{array}{cc} R_{\mathrm{dem}} & =-\kappa \cdot \mathrm{peak}, \end{array}$$

where

- *E* = $\sum_{t=0}^{H-1}$$u_{t}\Delta t$ is the total scheduled energy (kWh);
- $E_{*}$ is the baseline reference daily energy (kWh)
- PROD is the total daily production (ton);
- ${\mathrm{PROD}}_{\min}$ is the minimum acceptable production target (ton);
- $\lambda_{\mathrm{ener}}$ and $\lambda_{\mathrm{prod}}$ are terminal penalty weights;
- $\mathrm{peak}=\max_{t\in P}u_{t}$ is the maximum power during the peak tariff window;
- κ is the normalized demand charge coefficient (cost units/kW);
- *H* denotes the number of time steps in the episode;
- Δt is the time-step duration (1 h).

The total episodic return is therefore

(25) $$R=\sum_{t=0}^{H-1}r_{t}+R_{\mathrm{ener}}+R_{\mathrm{prod}}+R_{\mathrm{dem}}.$$

#### 3.3.8. State Representation

At each decision step *t*, the reinforcement learning agent observes a compact state vector defined as

(26) $$s_{t}=\left[\tau_{t},\;p_{t,\mathrm{norm}},\;b_{t,\mathrm{norm}},\;E_{t,\mathrm{norm}},\;{\mathrm{prod}}_{t,\mathrm{norm}}^{\mathrm{base}},\;{\mathrm{prod}}_{t,\mathrm{norm}}^{\mathrm{sofar}}\right]\in R^{6},$$

where $\tau_{t}=t/(H-1)$ denotes the normalized temporal progress within the finite scheduling horizon of length *H*. The term $p_{t,\mathrm{norm}}$ represents the normalized electricity price at time *t*, obtained by scaling the hourly tariff by its maximum value over the episode. The normalized baseline load $b_{t,\mathrm{norm}}$ corresponds to the expected power demand at time *t*, scaled by the maximum baseline demand over the horizon.

The cumulative scheduled energy up to time *t* is captured by

(27) $$E_{t,\mathrm{norm}}=\frac{\sum_{k=0}^{t-1}u_{k}}{E_{*}},$$

where $u_{k}$ denotes the scheduled power at time step *k* and $E_{*}$ is the total baseline daily energy requirement.

To explicitly account for production feasibility, two production-related components are included in the state. The term ${\mathrm{prod}}_{t,\mathrm{norm}}^{\mathrm{base}}$ denotes the normalized baseline production reference at time *t*, computed as the ratio between the baseline hourly production and the total baseline daily production. The cumulative executed production up to time *t* is represented by ${\mathrm{prod}}_{t,\mathrm{norm}}^{\mathrm{sofar}}$, defined as the ratio between the accumulated production and the minimum required daily production.

This six-dimensional state representation provides the agent with simultaneous information about temporal progress, price signals, baseline demand, cumulative energy usage, and production targets. As a result, the policy can make tariff-aware scheduling decisions while explicitly tracking progress toward daily energy balance and production feasibility constraints.

#### 3.3.9. Training Objective

The agent aims to learn a policy $\pi_{\theta }$ that maximizes the expected episodic return

(28) $$\max_{\theta }\;E\left[R\right].$$

Policy optimization is performed using Proximal Policy Optimization (PPO) [30], whose clipped surrogate objective is given by

(29) $$\begin{array}{cc} L_{\mathrm{PPO}}\left(\theta \right) & =E_{t}\left[\min\;\left(r_{t}\left(\theta \right)\;{\hat{A}}_{t},\;\mathrm{clip}\left(r_{t}\left(\theta \right),1-\epsilon ,1+\epsilon \right)\;{\hat{A}}_{t}\right)\right] \end{array}$$

(30) $$\begin{array}{cc} \; & \;-c_{v}{∥V_{\phi }\left(s_{t}\right)-{\hat{V}}_{t}∥}^{2}+c_{\mathrm{ent}}\;H\left(\pi_{\theta }\left(\cdot |s_{t}\right)\right), \end{array}$$

where $r_{t}\left(\theta \right)$ denotes the policy likelihood ratio.

#### 3.3.10. Key Performance Indicators

Performance is evaluated using the following indicators:

(31) $$\begin{array}{cc} \mathrm{kWh}/\mathrm{ton} & =\frac{\sum_{t}u_{t}}{\max(10^{-6},\mathrm{PROD})}, \end{array}$$

(32) $$\begin{array}{cc} \mathrm{Cost}/\mathrm{ton} & =\frac{C_{\mathrm{tot}}}{\max(10^{-6},\mathrm{PROD})}, \end{array}$$

(33) $$\begin{array}{cc} \mathrm{Peak}\;\mathrm{load}\;\mathrm{during}\;\mathrm{TOU} & =\max_{t\in P}u_{t}. \end{array}$$

It is important to note that the scheduling horizon considered in this work corresponds to a single industrial production shift (08:00–16:00), reflecting the operational structure of the studied quicklime production facility. Production activities are organized in daily shifts with fixed operational targets, while no production occurs during nighttime hours. Under these conditions, cross-day load transfer is not operationally feasible because postponing energy consumption to the following day would directly affect the planned daily production volume and kiln operation schedule.

Consequently, the proposed framework focuses on intra-shift load redistribution within the available operational window. This formulation aligns with typical day-ahead planning practices in industrial energy management systems. Extending the scheduling horizon to multi-day optimization, particularly in processes with intermediate storage or flexible batch production, represents a promising direction for future research.

#### 3.3.11. Time-of-Use Tariff Structure

The industrial electricity tariff considered in this study follows a time-of-use (TOU) structure during the operating shift (08:00–16:00). To preserve contractual confidentiality, all monetary values are reported in normalized tariff units rather than absolute currency values. The normalized hourly energy price $p_{t}$ (in cost units per kWh) is defined in Table 2 as follows:

The peak period is defined as the interval from 13:00 to 15:00, during which higher energy prices apply and demand exposure is penalized.

In addition to the volumetric energy charge, a demand charge component is included to account for peak power exposure during the defined peak window. The demand cost is computed as

(34) $$C_{\mathrm{dem}}=\kappa \cdot \max_{t\in P}u_{t},$$

where κ represents the normalized demand charge coefficient (cost units/kW), and P denotes the peak window (13:00–15:00).

Although the absolute monetary values are normalized, the tariff structure reflects the relative price differentials and demand-penalization mechanisms of a real medium-voltage industrial TOU contract applied in Colombia.

### 3.4. Tariff-Aware Scheduling via Reinforcement Learning

The proposed tariff-aware scheduling strategy is implemented as a reinforcement learning (RL) problem using a custom environment developed in Python and compliant with the Gymnasium interface. Each episode spans the 9 hourly decision steps of the industrial operating shift (08:00–16:00), corresponding to the active production period of the plant.

At each hourly time step *t*, the agent observes the state vector $s_{t}$ defined in Section 3 (state representation), which provides: (i) the normalized temporal progress within the shift, (ii) the normalized baseline production reference at time *t*, (iii) the normalized cumulative production achieved so far, and (iv) the normalized time-varying electricity tariff. This compact representation allows the policy to take tariff-aware decisions while explicitly tracking progress toward the daily production target.

The action space is discrete and consists of selecting a scaling factor applied to the baseline consumption at each hour, $a_{t}\in \left\{0.80,\;0.90,\;1.00,\;1.10,\;1.25\right\}$, constrained within the operational bounds [0.80,1.25]× the baseline. These limits reflect feasibility constraints in industrial operation, where a minimum load sustains production continuity and moderate short-term overloading may be acceptable.

The reward function balances electricity cost minimization with temporal feasibility and production preservation. At each step, the agent receives a negative reward proportional to the instantaneous electricity cost. A quadratic tracking term weighted by $\beta_{\mathrm{trk}}=0.6$ discourages excessive postponement of energy consumption by penalizing deviations between scheduled cumulative energy and the baseline cumulative trajectory. At the end of the episode, a quadratic terminal penalty weighted by $\lambda_{\mathrm{ener}}=15$ enforces compliance with the baseline daily energy requirement. To preserve production, a terminal quadratic penalty weighted by $\lambda_{\mathrm{prod}}=40$ is applied if the total daily production falls below 95% of the baseline production target.

After training, the learned policy tends to reduce exposure during the TOU peak window (13:00–15:00) by reallocating part of the load within the operating shift. In practice, the resulting cost reductions arise from a combination of (i) peak-exposure mitigation (lower peak-window energy and lower peak-window power peak, reducing the demand-charge component) and (ii) a cost–production trade-off under the current reward parametrization, which may lead to net reductions in total daily energy and production in a fraction of days. Therefore, the framework should be interpreted as a tariff-aware scheduling layer whose feasibility and cost–production balance depend on reward shaping, penalty weights, and explicit constraint handling.

### 3.5. Practical Implementation and Control Command

The proposed controller is a supervisory scheduling layer operating at hourly resolution. For each hour *t* of the operating shift, the PPO policy outputs a discrete action $a_{t}\in \left\{0.80,\;0.90,\;1.00,\;1.10,\;1.25\right\}$, which is implemented as an hourly aggregate load setpoint by scaling the baseline trajectory, as follows:

(35) $$u_{t}=\mathrm{clip}\left(a_{t}b_{t},\;0.80\;b_{t},\;1.25\;b_{t}\right).$$

In practical terms, $u_{t}$ is an hourly target for the plant-level demand (kW) at the point of common coupling, suitable for integration with an industrial energy management system (EMS) or supervisory operator scheduling. Importantly, the proposed action does not model explicit equipment-level ON/OFF switching: since sensing is aggregated at the PCC, the controller operates on the total plant demand and enforces relative scaling of the baseline (i.e., partial-capacity operation at the aggregate level). In deployment, this setpoint can be realized by operator/EMS dispatch (e.g., sequencing high-power operations within the shift) or by a supervisory controller that adjusts allowable operating levels of aggregated loads, without requiring additional equipment-level metering or intrusive modifications.

In this study, the policy is evaluated in an offline day-ahead manner: the schedule ${\left\{u_{t}\right\}}_{t=0}^{H-1}$ is computed for a given day using the baseline profile and tariff signal, and then assessed against the baseline via cost and feasibility indicators. This setup corresponds to an offline day-ahead scheduling evaluation at hourly resolution, rather than real-time closed-loop control.

### 3.6. Reinforcement Learning Environment and State Definition

The reinforcement learning environment is implemented using the Gymnasium framework and operates over a discrete-time horizon of length *H*. At each time step, the environment updates the cumulative production and energy tracking terms according to the selected action.

### 3.7. Training Configuration and Hyperparameters

The scheduling policy is trained using Proximal Policy Optimization (PPO), an on-policy actor–critic algorithm that employs a clipped surrogate objective to stabilize policy updates and Generalized Advantage Estimation (GAE) to reduce variance in advantage computation. PPO is particularly well suited for constrained industrial control problems due to its robustness and sample efficiency.

Both the policy and value functions are parameterized by a multilayer perceptron (MLP) with two hidden layers of 64 neurons each and rectified linear unit (ReLU) activations. The main training hyperparameters are summarized as follows: discount factor γ=0.99, rollout length $n_{\mathrm{steps}}=256$, batch size 256, learning rate $3\times 10^{-4}$, and clipping range ϵ=0.2. An entropy regularization term is included to promote exploration during training and prevent premature convergence to suboptimal scheduling strategies.

The reward function incorporates multiple weighted components reflecting industrial priorities. The energy tracking penalty weight is set to $\beta_{\mathrm{trk}}=0.6$, while the terminal penalty for deviations from the baseline daily energy requirement is weighted by $\lambda_{\mathrm{ener}}=15.0$. To explicitly enforce production feasibility, a soft reward proportional to hourly production is included, together with a terminal quadratic penalty applied when the total daily production falls below a minimum threshold defined as 95% of the baseline production. The corresponding penalty weight is set to $\lambda_{\mathrm{prod}}=40.0$.

In addition to energy costs, a demand charge proportional to the maximum scheduled power during the peak tariff window (13:00–15:00) is included, with a demand rate coefficient of κ=2000 (relative units). This term encourages the agent to reduce peak demand exposure in addition to minimizing energy costs.

Training is performed over multiple representative working days extracted from the measured industrial dataset, while performance evaluation is conducted on separate, non-overlapping days to assess the generalization capability of the learned scheduling policy.

Table 3 summarizes the reinforcement learning configuration and hyperparameters adopted in this study for tariff-aware load scheduling. The scheduling policy is trained using Proximal Policy Optimization (PPO) within a custom Gymnasium environment operating over a finite horizon of nine hourly decision steps corresponding to the industrial working shift. The table reports the structure of the state and action spaces, the neural network architecture used for the policy and value functions, and the main PPO training parameters, including discount factor, rollout length, batch size, learning rate, and clipping range. In addition, the key reward-related coefficients governing energy tracking, terminal energy balance, production feasibility, and peak-demand penalization are provided, ensuring full reproducibility of the proposed control strategy.

**Figure 2:**
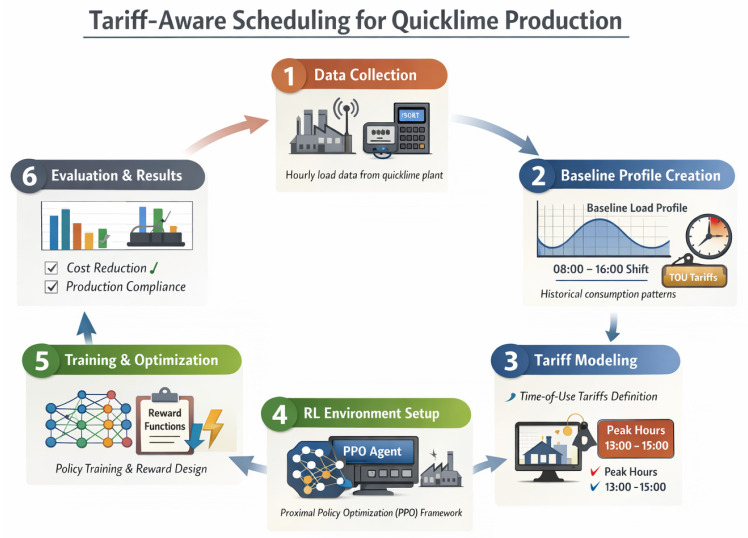
Overview of the tariff-aware reinforcement learning framework and experimental workflow.

Figure 2 provides a high-level overview of the proposed tariff-aware reinforcement learning framework and the complete experimental workflow followed in this study. The diagram summarizes the main stages required to reproduce the proposed approach, including data acquisition from an industrial energy monitoring system, construction of the baseline load profile, formulation of the tariff-aware reinforcement learning environment, training of the PPO-based scheduling policy, and multi-day performance evaluation under realistic operational constraints.

### 3.8. Computational Effort and Compute Cost

To support reproducibility and quantify the computational effort, we measured wall-clock runtimes for (i) daily schedule generation (inference/schedule construction) and, when applicable, (ii) the daily optimization cost. The scheduling horizon consists of nine hourly decisions (08:00–16:00). All measurements were obtained over N=30 validation days. The execution environment was Windows 10 with Python 3.10.13, an 8 C/16 T CPU, and 63.07 GB RAM; an RTX 5060 Ti GPU (16 GB) was available, but the reported measurements were run on CPU (torch was built without CUDA support in our setup).

For learning-based methods (PPO, DQN), we distinguish between: (i) training cost (offline) and (ii) per-day inference cost (generating the nine-step schedule). For DP, the cost corresponds to the per-day solve time, since the problem is explicitly re-optimized for each day. For GREEDY, the cost corresponds to the time required to construct the schedule using local rules.

The results in Table 4 show that, once trained, the learned policies (PPO/DQN) generate the daily schedule in the millisecond range, which is compatible with hourly-resolution day-ahead scheduling. In contrast, DP incurs a substantially higher computational cost because it must solve the optimization problem from scratch for each day. The GREEDY heuristic yields the lowest compute cost, as it relies on local rules to construct the schedule.

## 4. Results

### 4.1. Performance of the PPO Policy Under TOU Tariffs

The controller was evaluated using a PPO policy trained for tariff-aware scheduling under time-of-use (TOU) tariffs. The analysis focuses on: (i) temporal demand redistribution during the operating shift (08:00–16:00), (ii) its impact on total electricity cost (energy and demand charges), and (iii) the effect on operational indicators, particularly production and energy intensity.

#### 4.1.1. Representative Day

**Figure 3:**
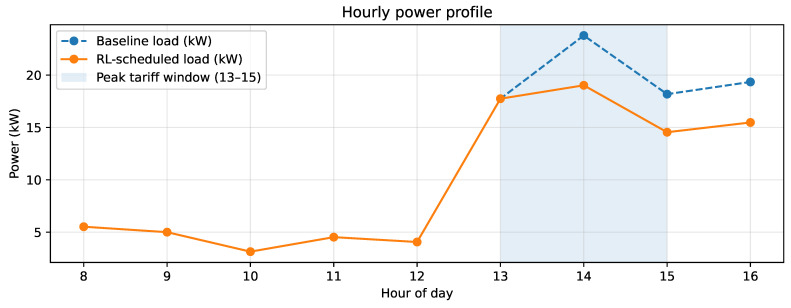
Hourly power profile during the operating shift (08:00–16:00): baseline demand versus the schedule obtained with the PPO policy. The shaded region highlights the TOU tariff window (13:00–15:00).

Figure 3 shows the hourly power profile for the representative day (7 February 2024), comparing baseline operation with the schedule produced by PPO. Baseline operation exhibits higher load exposure during the TOU window (13:00–15:00), whereas PPO reduces demand in that interval and reallocates part of the load to adjacent hours, consistent with tariff arbitrage aimed at lowering total cost.

Table 5 summarizes the representative-day indicators. PPO reduces total cost from 110,703.252 to 93,226.357 relative units (absolute savings of 17,476.895; 15.787%). This reduction is driven by concurrent decreases in the energy charge (63,133.336 to 55,170.422) and the demand charge (47,569.916 to 38,055.935), associated with lower peak-period exposure. Within the TOU window, peak-window energy decreases from 59.718 to 51.324 kWh and the peak-window power peak is reduced from 23.785 to 19.028 kW. However, the savings are accompanied by reductions in total daily energy (101.323 to 89.059 kWh) and daily production (0.248 to 0.213 ton), which increases energy intensity from 408.163 to 418.100 kWh/ton. Therefore, under the current reward parametrization, PPO prioritizes cost and peak-exposure reduction, with a noticeable production penalty on the analyzed day. Importantly, the observed savings are not explained solely by reduced output. The cost reduction is also linked to systematic mitigation of peak exposure during the TOU window: PPO lowers both peak-window energy and the peak power, which directly decreases the demand-charge component. At the same time, under the current reward parametrization, the policy may combine peak avoidance with a net reduction in total daily energy and production, evidencing a cost–production trade-off rather than pure load shifting.

#### 4.1.2. Multi-Day Validation (N=30)

To assess robustness beyond a single day, PPO was validated over N=30 days and compared against the baseline in terms of: (i) economic savings and total cost, (ii) peak-period exposure (energy and power peak), (iii) energy intensity (kWh/ton), and (iv) compliance flags reported by the evaluation script.

**Figure 4:**
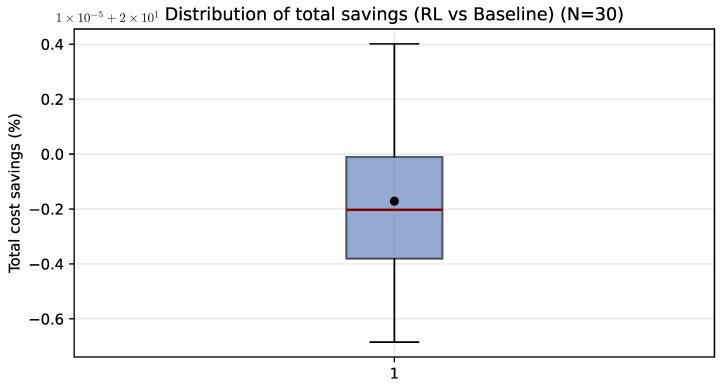
Distribution of daily percentage savings achieved by PPO relative to baseline over 30 validation days. The black dot represents the mean total cost savings across the 30 independent runs, while the red line inside the box indicates the median value.

Figure 4 highlights inter-day variability in percentage savings: the interquartile range and the presence of extreme values indicate that the economic benefit depends on how strongly each daily baseline profile is exposed to the TOU window. Descriptive statistics are summarized in Table 6.

**Figure 5:**
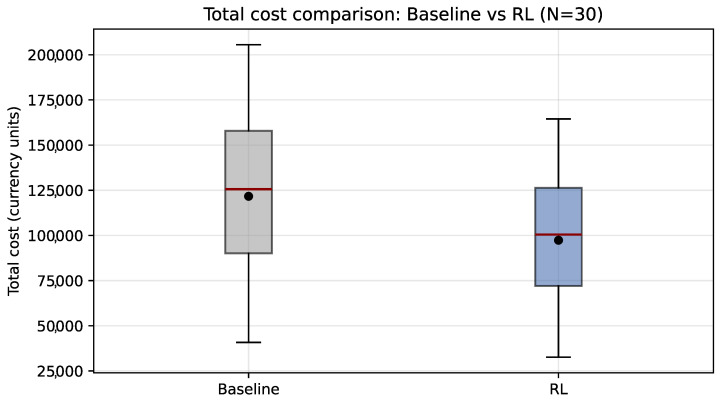
Boxplot comparison of daily total cost under baseline operation and PPO scheduling over 30 validation days. The black dot represents the mean total cost savings across the 30 independent runs, while the red line inside the box indicates the median value.

As shown in Figure 5, the daily total-cost distribution shifts toward lower values under PPO, suggesting a systematic cost reduction rather than an effect driven by a small number of atypical days. This behavior is consistent with reduced peak exposure (energy and peak power), summarized in Table 6.

**Figure 6:**
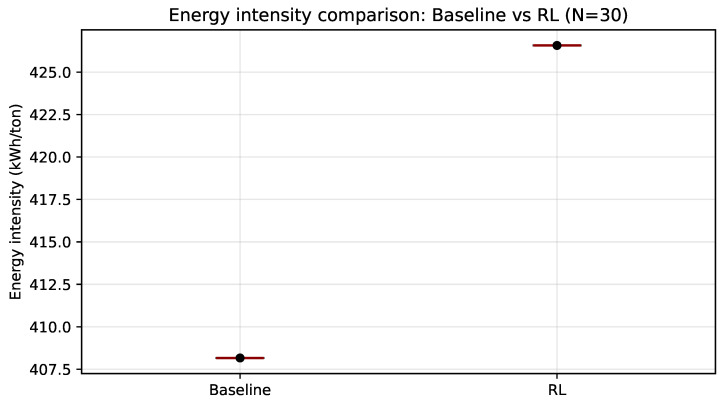
Comparison of energy intensity (kWh/ton) between baseline operation and PPO over 30 validation days. The black dot represents the mean total cost savings across the 30 independent runs, while the red line inside the box indicates the median value.

Figure 6 shows that, under the reference scheme, baseline energy intensity remains constant, whereas PPO yields a concentrated distribution (low dispersion) shifted toward higher values. Overall, this indicates that the policy prioritizes tariff-cost reduction and peak-demand mitigation, without strictly optimizing specific energy per produced ton under the current reward configuration (Table 6).

Across N=30 days, the reduction in total cost is consistent with a systematic decrease in peak-period exposure (both peak-window energy and peak power peak), indicating that a substantial portion of the benefit arises from reducing demand-charge exposure during the TOU window. Nevertheless, the increase in energy intensity relative to the baseline suggests that the savings are achieved under a trade-off between cost minimization and production preservation, which motivates stricter constraint handling and reward tuning when production feasibility must be guaranteed.

Finally, the evaluation script reports two daily binary indicators: viol_energy and viol_prod_min. The flag viol_energy equals 1 when the policy does not meet the defined daily energy-balance criterion (e.g., a relative deviation from the daily energy target exceeding a tolerance), and 0 otherwise. Similarly, viol_prod_min equals 1 when daily production falls below the prescribed minimum production level, and 0 when the requirement is satisfied. In this run, viol_energy was triggered in 28/30 days (rate 0.933) and viol_prod_min in 12/30 days (rate 0.400), suggesting that, under the selected thresholds, the policy frequently deviates from the energy-balance criterion and exhibits minimum-production violations in a smaller but non-negligible fraction of days. These results highlight the need to strengthen constraint handling (e.g., penalty weights and tolerances) when stricter operational guarantees are required.

The validation period comprises N=30 working days available from the industrial monitoring system. This window provides a multi-day backtesting assessment of the proposed scheduler under real plant operation and the observed TOU tariff structure; however, it does not necessarily represent the full 12-month operational cycle. In particular, longer-term variability may arise from changes in production intensity, planned maintenance shutdowns, process disruptions, and potential updates in tariff structures (energy and/or demand-charge components). Therefore, the reported savings should be interpreted as short-term evidence of consistent tariff-driven benefits within the monitored period, rather than as a direct estimate of annual savings.

A rigorous annual generalization would require extending the evaluation to a multi-month dataset spanning different operational regimes, and/or performing a scenario-based backtesting under alternative tariff schedules. This extension is left for future work and will enable quantifying the stability of savings under longer-term variability and operational interruptions.

### 4.2. Benchmarking Against Alternative Methods

To strengthen the experimental evaluation, PPO was compared with three alternative strategies: (i) a classical optimization reference based on dynamic programming (DP), (ii) an alternative RL policy based on DQN, and (iii) a low-complexity greedy heuristic (GREEDY). This comparison contextualizes performance in terms of economic savings and operational implications (energy and production), and distinguishes horizon-consistent methods from local decision rules.

#### 4.2.1. Deterministic Optimal Reference (DP)

On the representative day (7 February 2024), DP reduces total cost from 110,703.25 to 104,801.24 relative units (5.33%). This reduction is achieved by decreasing peak exposure (peak-window energy: 59.718 to 57.383 kWh; peak-window power peak: 23.785 to 21.406 kW) with moderate production impact (Δproduction ≈−2.09%). In multi-day validation (N=30), DP achieves average savings of 9.302 ± 6.314% and exhibits low computation time (0.025 ± 0.017 s per day), providing a solid and efficient reference for model-based daily scheduling.

Regarding compliance (as per the script flags), DP reports rates of 0.90 for viol_energy and 0.20 for viol_prod_min. Since these metrics depend on specific tolerances, their interpretation should be supported by explicit definitions of the thresholds implemented.

#### 4.2.2. Alternative RL Policy (DQN)

On the representative day (7 February 2024), DQN achieves the highest economic savings (20.00%), but does so through a marked reduction in both total energy (−20.00%) and production (−23.45%), increasing cost per ton and degrading energy intensity (426.569 kWh/ton). In multi-day validation, DQN maintains high savings but exhibits systematic violations of energy and minimum production constraints (rate 1.00 for both flags), indicating that its economic performance is driven by an aggressive curtailment strategy not aligned with operational feasibility criteria.

#### 4.2.3. Greedy Heuristic (GREEDY)

The GREEDY heuristic is used as a low-complexity benchmark based on a local decision rule aimed at reducing hourly cost. On the representative day (7 February 2024), GREEDY reduces total cost by 3.43% while slightly increasing total energy (+0.12%) and production (+1.05%). This suggests that the observed savings arise from local redispatch and net energy variations. Since GREEDY does not enforce global energy/production constraints, it should not be interpreted as optimal or directly comparable to horizon-constrained approaches, but rather as a simple reference for benefits achievable with deterministic rules.

#### 4.2.4. Comparative Synthesis

Overall, the results reveal distinct trade-offs between economic savings and operational feasibility (Table 7). DQN attains the highest savings on the representative day (20.00%) and preserves a high-savings pattern in multi-day validation; however, it achieves these savings through aggressive energy and production curtailment (ΔE≈−20.00%, Δproduction ≈−23.45%), which degrades cost per ton and is accompanied by systematic constraint violations (viol_energy = 1.00; viol_prod_min = 1.00). Consequently, DQN is best interpreted as a reference illustrating an extreme cost-driven policy, rather than a viable alternative under strict industrial energy and production targets.

DP provides a classical, conservative, and computationally efficient deterministic reference. On the representative day, it delivers more moderate savings (5.33%) with limited production impact (Δproduction ≈−2.09%) while reducing peak exposure. In multi-day validation, DP achieves consistent savings at low computation time, but it requires an explicit model and solving the scheduling problem for each scenario, which can limit flexibility as operating conditions or constraints evolve.

The GREEDY heuristic is included as a low-complexity benchmark and shows that simple local rules can capture a fraction of the tariff benefit (3.43% on the representative day). However, because it does not enforce global energy/production constraints, it may alter the daily balance and cannot guarantee horizon-level feasibility; therefore, it is primarily illustrative and does not replace constraint-aware methods.

Finally, PPO offers a competitive compromise between savings and peak-demand mitigation through a learned policy that is reusable at inference time. On the representative day, PPO achieves high savings (15.787%) with a smaller production reduction than DQN (Δproduction ≈−14.11% versus −23.45%), which is reflected in a more favorable savings–production trade-off (Table 7). In multi-day validation, PPO maintains consistent economic benefits and systematic peak-exposure reductions, with a lower minimum-production violation rate than DQN, albeit higher than DP. Overall, these results position PPO as a competitive alternative for TOU-aware scheduling, particularly when the operational objective is to reduce cost and peak demand while retaining the ability to tune the cost–production balance via reward parametrization and/or stricter constraint handling.

## 5. Conclusions

This work evaluated four tariff-aware scheduling strategies for an aggregated energy-intensive industrial process under a unified TOU tariff framework: a reinforcement learning policy based on Proximal Policy Optimization (PPO), a deterministic dynamic programming (DP) benchmark, a Deep Q-Network (DQN) policy, and a low-complexity greedy heuristic.

The comparison highlights distinct trade-offs. DP provides stable and computationally efficient cost reductions but requires explicit daily re-optimization under changing conditions. DQN can achieve large nominal savings, yet often through aggressive energy and production curtailment, leading to degraded operational indicators. The greedy heuristic captures limited tariff benefits but lacks horizon consistency and constraint awareness.

Within this context, PPO offers the most balanced compromise between cost reduction, peak-demand mitigation, and deployment flexibility. On both the representative day and the 30-day validation window, PPO systematically reduced total cost and peak-period exposure relative to baseline operation. The savings primarily arise from tariff-aware demand management within the operating shift.

However, the results also reveal a trade-off between economic optimization and strict preservation of operational metrics. Under the current reward configuration, PPO may produce moderate reductions in total energy and production, occasionally triggering feasibility flags. This underscores the importance of transparent compliance definitions and stronger constraint handling when stricter guarantees are required.

It should be noted that the evaluation period comprises 30 monitored working days; therefore, the reported savings represent short-term evidence and should not be directly extrapolated to annual operation without multi-month validation under varying production and tariff conditions.

The proposed framework operates on aggregated measurements at the point of common coupling, facilitating integration with existing industrial energy management systems without requiring equipment-level instrumentation. Future work will focus on enhanced constraint enforcement, multi-day scheduling extensions, and uncertainty-aware learning strategies for realistic tariff and load variability.

## Figures and Tables

**Table 1:** Main electrically driven equipment in the SUMININCO quicklime production line.

Process Stage	Equipment	Motor Power	Load Type
Material feeding	Hopper 1	20 HP	AC motor (material flow control)
Primary crushing	Crusher	30 HP	AC motor (size reduction)
Material transport	Belt conveyor 1	3 HP	Gear motor (continuous transport)
Screening	Vibrating sieve	25 HP	AC motor (particle size separation)
Material transport	Belt conveyor 2	3 HP	AC motor (material transfer)
Vertical transport	Bucket elevator	3 HP	AC motor (material lifting)
Material transport	Belt conveyor 3	3 HP	AC motor (furnace feed)
Furnace feeding system	Sky-type elevator belt	2 HP	AC motor (material lifting)
Furnace air draft system	Induced draft fan	–	Industrial fan (intermittent operation)
Furnace discharge	Lime outlet conveyor	2 HP	AC motor (material discharge)
Final crushing	Toothed roller mill	11 HP	AC motor (final size reduction)

**Table 2:** Normalized TOU tariff structure for the 08:00–16:00 operating shift.

Hour	Period	Normalized Price (Cost Units/kWh)
08:00–13:00	Off-peak	$p_{\mathrm{off}}$
13:00–15:00	Peak	$p_{\mathrm{peak}}$
15:00–16:00	Off-peak	$p_{\mathrm{off}}$

**Table 3:** Reinforcement learning configuration and hyperparameters used for tariff-aware scheduling.

Category	Value/Description
RL algorithm	Proximal Policy Optimization (PPO)
Environment	Custom Gymnasium environment
Decision horizon	H=9 time steps (08:00–16:00)
State dimension	6 (normalized time, price, baseline load,
	cumulative energy, baseline production,
	cumulative production)
Action space	Discrete scaling factors
	{0.80,0.90,1.00,1.10,1.25}
Load bounds	[0.80,1.25]× baseline load
Policy network	MLP with two hidden layers (64, 64)
Activation function	ReLU
Discount factor (γ)	0.99
Rollout length ($n_{\mathrm{steps}}$)	256
Batch size	256
Learning rate	$3\times 10^{-4}$
Clipping range (ϵ)	0.2
Entropy regularization	Enabled
Energy tracking weight ($\beta_{\mathrm{trk}}$)	0.6
Terminal energy penalty ($\lambda_{\mathrm{ener}}$)	15.0
Production penalty ($\lambda_{\mathrm{prod}}$)	40.0
Minimum production constraint	95% of baseline production
Demand charge coefficient (κ)	2000 (relative units)
Training data	Multiple representative working days
Evaluation strategy	Multi-day validation on unseen days

**Table 4:** Measured computational effort for N=30 days (9-decision horizon: 08:00–16:00).

Method	Training (s)	Time per Day (s)	Median (IQR) (s)	Interpretation
PPO	2.461 (15,000 steps)	0.002568 ± 0.000290	–	Inference (9-step schedule)
PPO	–	0.003182 ± 0.000196	–	Baseline + PPO evaluation (2 rollouts/day)
DQN	–	0.001495 ± 0.000045	0.001491 (0.001461–0.001520)	Inference (9-step schedule)
GREEDY	–	0.000210 ± 0.000048	0.000212 (0.000171–0.000237)	Schedule construction (rule-based)
DP	–	7.700915 ± 0.108250	7.708162 (7.615494–7.758799)	Daily solve (re-optimizes per day)

**Table 5:** Comparison of indicators between baseline operation and the PPO policy on a representative day (7 February 2024).

Metric	Baseline	PPO
Total energy consumption (kWh)	101.323	89.059
Total production (ton)	0.248	0.213
Energy intensity (kWh/ton)	408.163	418.100
Peak-window energy (kWh)	59.718	51.324
Peak-window power peak (kW)	23.785	19.028
Energy charge (relative units)	63,133.336	55,170.422
Demand charge (relative units)	47,569.916	38,055.935
Total cost (relative units)	110,703.252	93,226.357
Cost per ton (units/ton)	445,950.649	437,663.383

**Table 6:** Statistical summary of PPO performance over 30 validation days (compared with baseline).

Metric	Mean ± Std. Dev.	Median (IQR)
Total savings (%)	9.802 ± 4.885	11.933 (5.503–13.252)
Absolute savings (relative units)	12,873.439 ± 8370.734	14,888.601 (4456.721–18,865.625)
Baseline total cost (relative units)	121,669.616 ± 45,617.340	125,616.751 (90,065.356–157,945.442)
PPO total cost (relative units)	108,796.178 ± 39,137.674	116,883.919 (79,527.356–137,976.619)
Baseline energy intensity (kWh/ton)	408.163 ± 0.000	408.163 (408.163–408.163)
PPO energy intensity (kWh/ton)	413.985 ± 2.658	414.017 (412.633–415.586)
Baseline peak-window energy (kWh)	48.519 ± 20.700	55.024 (31.993–63.711)
PPO peak-window energy (kWh)	43.006 ± 16.970	47.659 (29.150–54.434)
Baseline peak-window power peak (kW)	20.472 ± 8.689	21.652 (15.793–27.782)
PPO peak-window power peak (kW)	17.151 ± 6.517	18.658 (15.174–22.226)

Representativeness of the 30-day window.

**Table 7:** Performance comparison on the representative day (7 February 2024) and in multi-day validation (N=30). GREEDY is reported only for the representative day. viol_energy and viol_prod_min are daily binary flags. Trade-off = Savings%/|ΔProduction%|.

Metric	Baseline	PPO	DP	DQN	GREEDY
Representative day (7 February 2024)					
Total savings (%)	0.00	15.787	5.33	20.00	3.43
Total cost (r.u.)	110,703.252	93,226.357	104,801.24	88,562.61	106,908.500
Total energy (kWh)	101.323	89.059	99.388	81.058	101.443
Δ Total energy (%)	0.00	−12.10	−1.91	−20.00	+0.12
Total production (ton)	0.248	0.213	0.243052	0.190	0.250840
Δ Production (%)	0.00	−14.11	−2.09	−23.45	+1.05
Energy intensity (kWh/ton)	408.163	418.100	408.916	426.569	404.414
Peak-window energy (kWh)	59.718	51.324	57.383	47.775	55.762
Peak-window power peak (kW)	23.785	19.028	21.406	19.028	22.186
Cost per ton (r.u./ton)	445,950.649	437,663.383	431,187.86	466,060.36	426,202.101
Trade-off savings/|Δprod| (—)	–	1.12	2.55	0.85	–
Multi-day validation (N=30)					
Total savings (%) (mean ± SD)	–	9.802 ± 4.885	9.302 ± 6.314	20.000 ± 0.000	–
Total savings (%) (median, IQR)	–	11.933 (5.503–13.252)	8.142 (6.918–9.288)	20.000 (20.000–20.000)	–
viol_energy (rate)	–	0.933	0.90	1.00	–
viol_prod_min (rate)	–	0.400	0.20	1.00	–
Computation/inference time (s)	–	–	0.025 ± 0.017	0.001 ± 0.000	–

## Data Availability

The data and code of this research are available in this web page: https://github.com/jerssonleon/reinforcement-learning-energy-scheduling (accessed on 24 January 2026).

## Abbreviations

The following abbreviations are used in this manuscript:

RL	Reinforcement Learning
DRL	Deep Reinforcement Learning
PPO	Proximal Policy Optimization
TOU	Time-of-Use
GAE	Generalized Advantage Estimation
MLP	Multilayer Perceptron
EV	Expected Value
kW	Kilowatt
kWh	Kilowatt-Hour
